# Echinocandin Drugs Induce Differential Effects in Cytokinesis Progression and Cell Integrity

**DOI:** 10.3390/ph14121332

**Published:** 2021-12-20

**Authors:** Natalia Yagüe, Laura Gómez-Delgado, M. Ángeles Curto, Vanessa S. D. Carvalho, M. Belén Moreno, Pilar Pérez, Juan Carlos Ribas, Juan Carlos G. Cortés

**Affiliations:** Instituto de Biología Funcional y Genómica, Consejo Superior de Investigaciones Científicas (CSIC) and Universidad de Salamanca, 37007 Salamanca, Spain; natalia98@usal.es (N.Y.); laugomdel@usal.es (L.G.-D.); emecur@usal.es (M.Á.C.); vanessa.sdc@usal.es (V.S.D.C.); mbms@usal.es (M.B.M.); piper@usal.es (P.P.); ribas@usal.es (J.C.R.)

**Keywords:** fungi, invasive fungal infections, fission yeast, cell wall, β(1,3)-D-glucan synthase, antifungal drugs, echinocandin drugs, echinocandin resistance, Fks resistance hot spots, cytokinesis, septation, cell separation, cell integrity, cell lysis

## Abstract

Fission yeast contains three essential β(1,3)-D-glucan synthases (GSs), Bgs1, Bgs3, and Bgs4, with non-overlapping roles in cell integrity and morphogenesis. Only the *bgs4^+^* mutants *pbr1-8* and *pbr1-6* exhibit resistance to GS inhibitors, even in the presence of the wild-type (WT) sequences of *bgs1^+^* and *bgs3^+^*. Thus, Bgs1 and Bgs3 functions seem to be unaffected by those GS inhibitors. To learn more about echinocandins’ mechanism of action and resistance, cytokinesis progression and cell death were examined by time-lapse fluorescence microscopy in WT and *pbr1-8* cells at the start of treatment with sublethal and lethal concentrations of anidulafungin, caspofungin, and micafungin. In WT, sublethal concentrations of the three drugs caused abundant cell death that was either suppressed (anidulafungin and micafungin) or greatly reduced (caspofungin) in *pbr1-8* cells. Interestingly, the lethal concentrations induced differential phenotypes depending on the echinocandin used. Anidulafungin and caspofungin were mostly fungistatic, heavily impairing cytokinesis progression in both WT and *pbr1-8*. As with sublethal concentrations, lethal concentrations of micafungin were primarily fungicidal in WT cells, causing cell lysis without impairing cytokinesis. The lytic phenotype was suppressed again in *pbr1-8* cells. Our results suggest that micafungin always exerts its fungicidal effect by solely inhibiting Bgs4. In contrast, lethal concentrations of anidulafungin and caspofungin cause an early cytokinesis arrest, probably by the combined inhibition of several GSs.

## 1. Introduction

Owing to the increasing population of immunocompromised patients, life-threatening systemic fungal infections have become a major risk for public health. A weakened immune system because of illnesses, such as cancer, diabetes, or HIV/AIDS syndrome, together with the practice of treatments or medicines, such as chemotherapy, organ transplantation, or corticoids, might predispose the afflicted to secondary fungal infections [1,2,3]. Thus, it has been estimated that over 1.6 million people die each year because of severe fungal infections [1,4]. Only four classes of antifungal drugs (echinocandins, polyenes, pyrimidine analogues, and triazoles) are used for treating systemic fungal infections [5,6,7]. Thus, the problem of systemic fungal infections is also worsened by the emergence of fungi resistant to one or several classes of the available antifungals [8,9].

The cell wall is a structure external to the plasma membrane present in all fungal cells. Its integrity is crucial for fungal survival, making the boundary between the cytoplasm and the external milieu and maintaining the internal turgor pressure [10,11]. Because the cell wall is not present in the infected animal hosts, molecules that inhibit its synthesis are appealing as potential antifungal drugs [12,13]. Structural microfibrils of the β(1,3)-D-glucan polysaccharide are the most abundant components in the cell wall framework [14,15,16]. The fission yeast *Schizosaccharomyces pombe* is an attractive model for exploring the role of the β(1,3)-D-glucan in cell wall synthesis, morphogenesis, and cell integrity. The *S. pombe* cell wall has no detectable amounts of chitin [16,17,18]; instead, it contains three different and essential β-glucans: a major branched β(1,3)-D-glucan that is the main responsible for the cell wall structure and integrity; a minor linear β(1,3)-D-glucan, which is responsible for the primary septum structure; and a minor branched β(1,6)-D-glucan [19,20].

In all the studied fungi, the catalytic subunit of the β(1,3)-D-glucan synthase (GS), the glycosyltransferase in charge of the β(1,3)-D-glucan synthesis, is constituted by the family of integral membrane proteins Bgs/Fks [19,21,22]. *S. pombe* contains four essential GS catalytic subunits: Bgs1 synthesizes the linear β(1,3)-D-glucan of the primary septum [19] and is required for septum ingression [19,23,24,25]; Bgs2 is essential for spore wall maturation [26,27]; Bgs3 is essential, although its function remains unknown [28]; and Bgs4 is the only subunit that has been shown to form part of the GS enzyme. It synthesizes the branched β(1,3)-D-glucan and is responsible for most GS activity [19,29]. The branched β(1,3)-D-glucan produced by Bgs4 is vital for maintaining cell shape and septum formation and completion. Differently from Bgs1 and Bgs3, the function of Bgs4 is essential for cell integrity, and thus, Bgs4 depletion causes cell lysis and cytoplasm leakage in the growing poles and mainly in the medial region at the onset of septum degradation and cell separation [30].

Treatment with inhibitors of the GS blocks the incorporation of novel β(1,3)-D-glucan to the wall surrounding the sites of active growth and causes osmotic fragility and cell lysis in exponentially growing cells. Thus, treated fungal cells die by the focalized rupture of the underneath plasma membrane and the subsequent cytoplasm leakage [31,32,33,34]. Three families of antifungals, lipopeptides (echinocandins), acidic terpenoids (enfumafungin), or glycolipids (papulacandins), have been described to alter the integrity of the cell wall by targeting the GS [10,13,35]. From all of them, only the echinocandins anidulafungin, caspofungin, and micafungin have been authorized as drugs for treating systemic fungal infections (Table 1) [5,6,13,35]. Thus, because of their fungicidal effect in yeasts, echinocandins are proposed as the forefront treatment for *Candida albicans* infections [36,37].

Resistance to GS inhibitors is well conserved in fungi and is generally associated with point mutations located in highly conserved short regions (hot spots) of the Bgs/Fks proteins [29,38,39,40]. In *S. pombe*, *pbr1-8* and *pbr1-6* are the only isolated mutants exhibiting resistance to specific GS inhibitors [29]. These two mutants are exclusively due to point mutations in the *bgs4^+^* sequence. A single mutation within *bgs4^+^* confers resistance to specific GS inhibitors in the presence of the wild-type (WT) sequences of *bgs1^+^* and *bgs3^+^*, suggesting that the function of the two encoding synthases, Bgs1 and Bgs3, is not inhibited by the available GS inhibitors [29]. The mode of action of echinocandins inhibits the synthesis of the β(1,3)-D-glucan through a noncompetitive inhibition of the GS activity [41,42,43]. However, the integral membrane GS catalytic subunit has not been homogenously purified, and, thus, neither the biochemistry and structure of the GS complex, nor the molecular mechanism of echinocandin inhibition of the GS activity, have been entirely elucidated [22,44,45,46].

The absence of cell wall chitin and the presence of three essential GS catalytic subunits exhibiting both differential antifungal susceptibility and non-redundant roles in β(1,3)-D-glucan synthesis render *S. pombe* an ideal tool for studying echinocandins mechanism of action and resistance [19,29]. Bgs proteins are essential for different aspects of cytokinesis and cell integrity. Thus, we have compared the effect of several echinocandins in these cellular processes. For this purpose, *S. pombe* WT and *pbr1-8* strains were imaged through time-lapse fluorescence microscopy in the presence of the drugs. In trying to minimize the effects of cell wall compensatory mechanisms that might alter the echinocandins-derived phenotypes [47,48], time-lapses were performed at very early times of treatment (up to 3 h) with both sublethal (non-inhibitory) and lethal (inhibitory) concentrations of anidulafungin, caspofungin, and micafungin. Our study shows that, depending on the strain and the used concentration, the echinocandin drugs differentially affected the cytokinesis progression and cell integrity. Sublethal and lethal concentrations of micafungin selectively inhibited Bgs4, suggesting that Bgs1 and Bgs3 are not susceptible to this antifungal. Similarly, sublethal concentrations of anidulafungin and caspofungin primarily affected Bgs4, confirming that Bgs4 is the GS subunit most susceptible to echinocandins. Remarkably, lethal concentrations of anidulafungin and caspofungin severely affected cytokinesis progression, showing that besides their effect on Bgs4 activity, they also likely affect the function of Bgs1 and/or Bgs3.

## 2. Results and Discussion

### 2.1. Susceptibilities of WT and pbr1-8 Strains to Echinocandins

**Figure 1:**
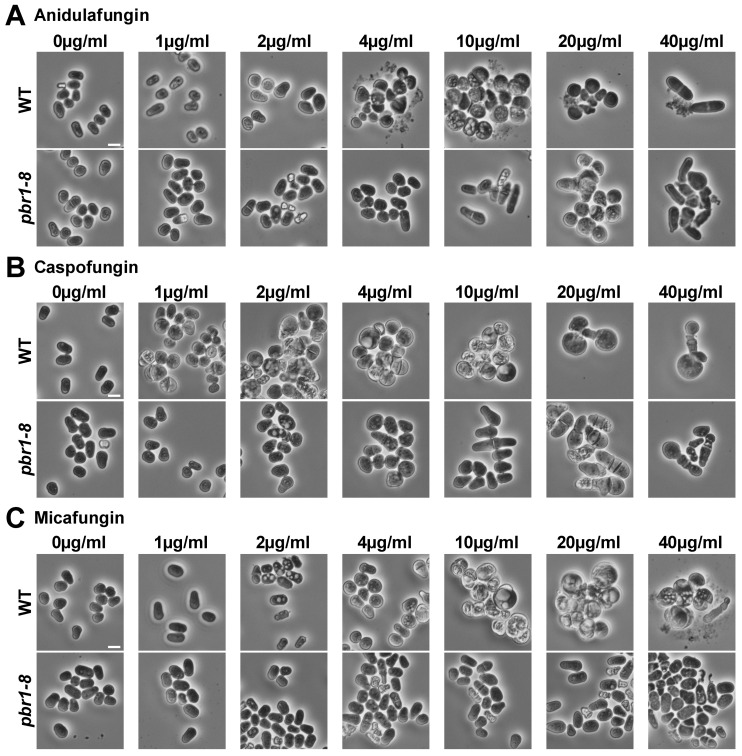
Morphology of WT and *pbr1-8* cells after 24 h of growth in the presence of increasing concentrations of anidulafungin (**A**), caspofungin (**B**), and micafungin (**C**) drugs. Early logarithmic-phase cells of the WT and *pbr1-8* strains growing in YES liquid medium at 28 °C were diluted to a high cell density of 5 × 10^6^ in micro-cultures of YES liquid medium containing either DMSO (0.8%, control) or increasing concentrations (1, 2, 4, 10, 20, and 40 µg/mL) of the drugs, grown with shaking for 24 h and imaged by phase-contrast microscopy. The data of this figure are developed in Table 2. Scale bars, 10 µm.

The susceptibility of yeasts to echinocandins depends on the number of inoculated cells. Thus, greater cell densities require higher drug concentrations to inhibit organism growth [41]. Because the imaging and preparation of yeast cells for time-lapse fluorescence microscopy requires cultures containing a higher density of cells than cultures used for visualizing the presence or absence of growth in susceptibility assays, we compared the susceptibilities to echinocandins of the WT and *pbr1-8* strains in cultures inoculated either with a typical lower cell density (5 × 10^5^ cells/mL) or with a higher cell density (5 × 10^6^ cells/mL) that will later be used in time-lapse fluorescence microscopy experiments (Table 2). The minimal inhibitory concentration (MIC) for the WT strain varied between 1 and 10 µg/mL at lower cell densities (Table 2, left column). As expected, the MIC for the WT increased to 10–20 µg/mL at higher cell densities (Table 2, right column). Thus, based on the obtained MICs with a higher number of cells, sublethal (non-inhibitory) and lethal (inhibitory) concentrations for the WT of 2 and 20 µg/mL, respectively, were chosen for performing the comparative time-lapse experiments (see next section). The *pbr1-8* strain exhibited a complete resistance to micafungin that was independent of the density of cells in the culture (a MIC of more than 80 µg/mL). In contrast, this strain presented some susceptibility to caspofungin and anidulafungin, with the MICs varying from 2–10 (lower cell density, Table 2, left column) to 20–40 µg/mL (higher cell density, Table 2, right column). Finally, micro-cultures starting at a higher cell density were used to examine the cell morphology by phase-contrast microscopy after 24 h of growth in the presence of increasing concentrations of the echinocandins. The three echinocandins led to the WT cells becoming aggregated, rounded, and swollen. This phenotype started to be observed in cells treated with sublethal concentrations of 1 µg/mL for caspofungin and 4 µg/mL for both anidulafungin and micafungin (Figure 1, WT). None of the applied concentrations of the micafungin was sufficient to induce the phenotype of swollen and rounded cells in the *pbr1-8* resistant strain; however, lethal concentrations of anidulafungin and caspofungin were able to induce it. (Figure 1, *pbr1-8*). These results show that micafungin selectively inhibits the function of Bgs4, whereas Bgs1 and Bgs3 are not affected by this antifungal. The same results were previously described for the other two classes of GS inhibitors, the glycopeptide papulacandin B and the acidic terpenoid enfumafungin that also selectively inhibited Bgs4 [29].

### 2.2. Differential Effects of the Echinocandins in Cytokinesis Progression

**Figure 2:**
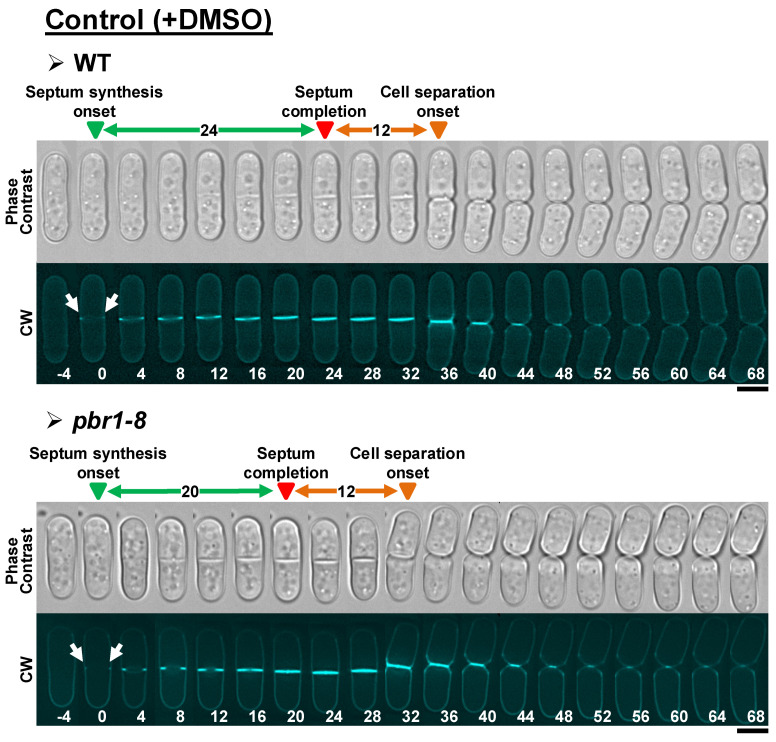
Normal cytokinesis (septum synthesis and cell separation) in WT and *pbr1-8* cells growing in the absence of the three echinocandin drugs. Early logarithmic-phase cells of the indicated strains growing in YES liquid medium at 28 °C were diluted to a cell density of 5 × 10^6^ in liquid YES medium containing both calcofluor white (CW, 1.25 µg/mL) and DMSO (0.8%), and then imaged by time-lapse fluorescence microscopy (1 medial z slice, 4 min elapsed time) for 3 h, as described in the Materials and Methods section. The data of this figure are developed in Table 3, Table 4 and Table 5. White arrow: first CW-stained septum synthesis. Arrowheads: blue, septum synthesis onset (time 0 for the elapsed time until septum synthesis completion); red, septum completion onset (time 0 for the elapsed time until cell separation onset); orange, cell separation onset. Scale bars, 5 µm.

**Figure 3:**
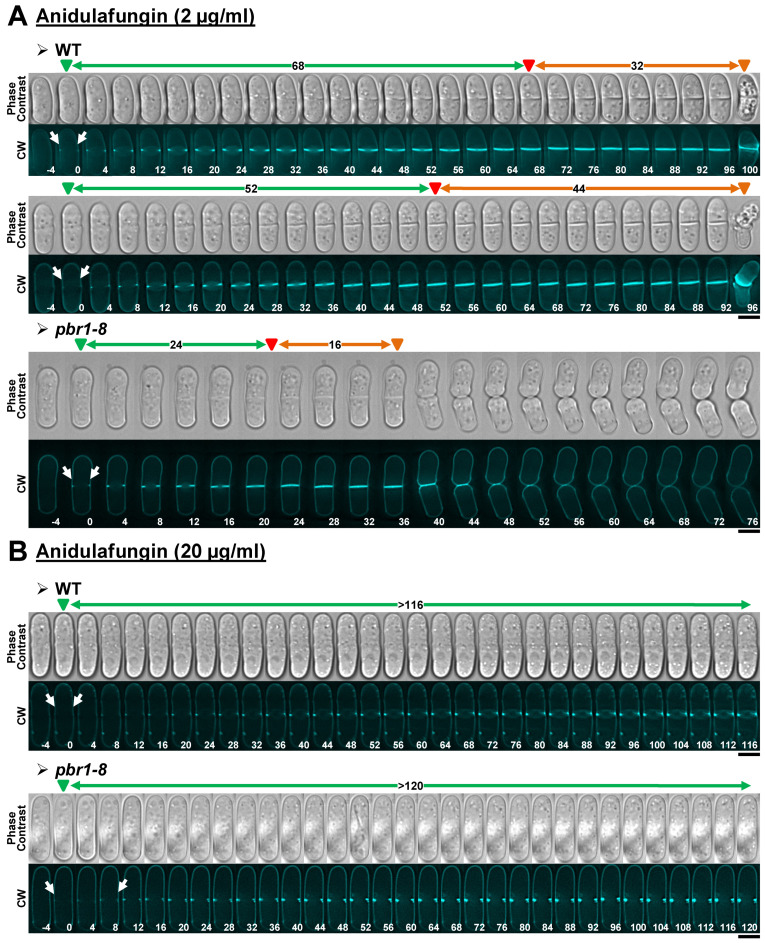
Cytokinesis phenotypes in WT and *pbr1-8* cells growing in the presence of sublethal and lethal concentrations of anidulafungin. The indicated strains were grown and imaged as in Figure 2 in the presence of either sublethal ((**A**), 2 µg/mL) or lethal ((**B**), 20 µg/mL) concentrations of the drug. The data of this figure are developed in Table 3, Table 4 and Table 5. Arrows and arrowheads are as in Figure 2. Scale bars, 5 µm.

**Figure 4:**
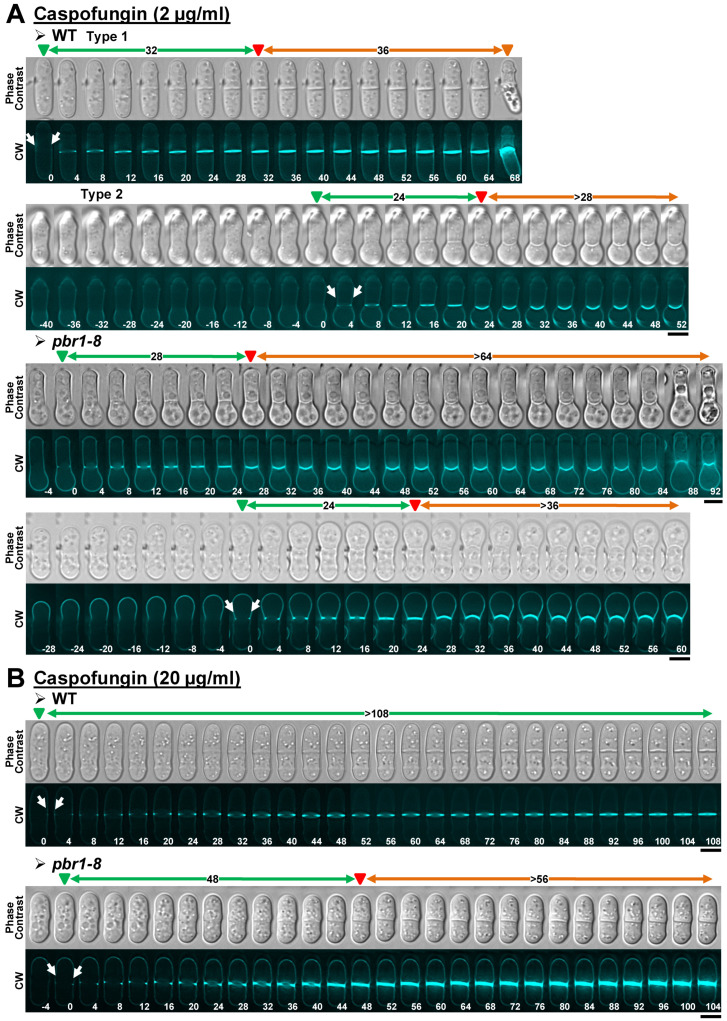
Cytokinesis phenotypes in WT and *pbr1-8* cells growing in the presence of sublethal and lethal concentrations of caspofungin. The indicated strains were grown and imaged as in Figure 2 in the presence of sublethal ((**A**), 2 µg/mL) or lethal ((**B**), 20 µg/mL) concentrations of the drug. The data of this figure are developed in Table 3, Table 4 and Table 5. Arrows and arrowheads are as in Figure 2. Scale bars, 5 µm.

**Figure 5:**
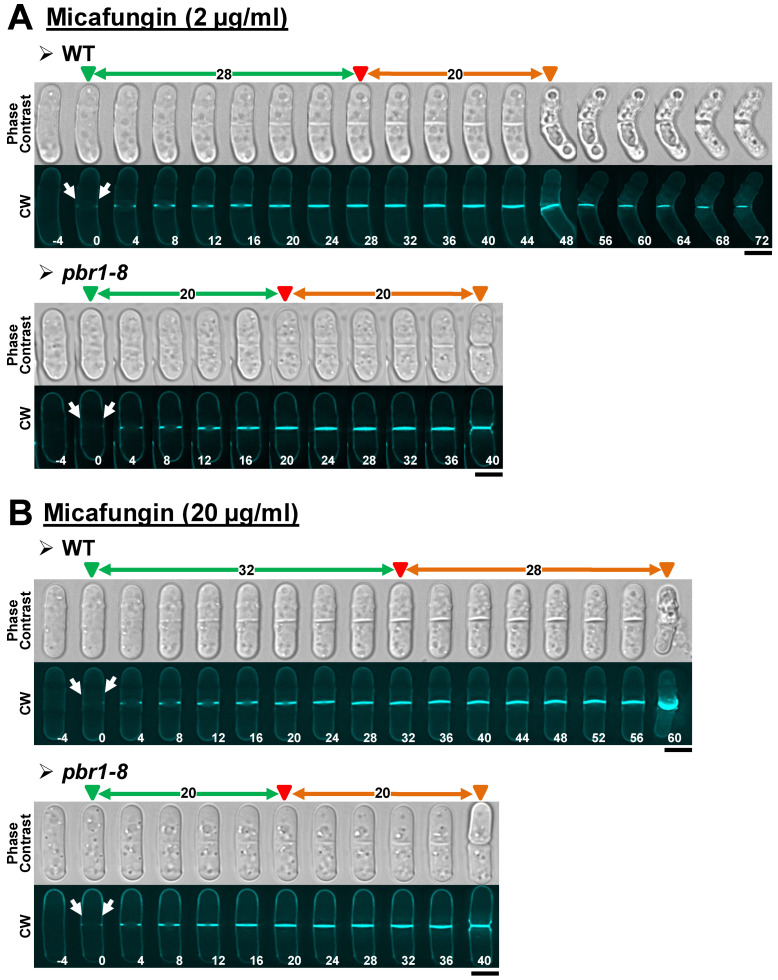
Cytokinesis phenotypes in WT and *pbr1-8* cells growing in the presence of sublethal and lethal concentrations of micafungin. The indicated strains were grown and imaged as in Figure 2 in the presence of sublethal ((**A**), 2 µg/mL) or lethal ((**B**), 20 µg/mL) concentrations of the drug. The data of this figure are developed in Table 3, Table 4 and Table 5. Arrows and arrowheads are as in Figure 2. Scale bars, 5 µm.

Cytokinesis is the last event of the cell cycle, where a cleavage furrow partitions the cell giving rise to two independent cells. In fungi, the ingression of the cleavage furrow is tightly coupled to the synthesis of a specialized cross wall named division septum [19,25,30,49]. In contrast to the filamentous fungi, yeast cytokinesis also requires the separation of daughter cells through the enzymatic degradation of the septum. To avoid cell lysis during cell separation, strict coordination between degradation and synthesis of the cell wall surrounding the septum is needed [30,50]. Thus, defects in the synthesis of the main cell wall glucans frequently lead to excessive wall degradation and cell lysis at the onset of cell separation [30]. Here, time-lapse fluorescence microscopy was used to examine the dynamics of septation and cell separation in the WT and *pbr1-8* strains during the first 3 h of echinocandin treatment. To follow the cytokinesis progression, cells were stained with very low doses of calcofluor white (CW), a fluorochrome that specifically and with high affinity binds to the linear β(1,3)-D-glucan of the primary septum, thus allowing the monitoring of cytokinesis since the very early steps of primary septum synthesis [49]. As described above, cells of the two strains were treated with both sublethal (2 µg/mL) and lethal (20 µg/mL) concentrations of the three echinocandins. As a control for the experiments, we first compared the cytokinesis progression in the WT and *pbr1-8* cells growing in the absence of echinocandins (control, Figure 2), observing that septum progression (double arrow depicted in green) and cell separation onset (double arrow depicted in orange) were similar in both strains. Table 3 compiles the data from the performed time-lapse microscopy experiments, showing the elapsed minutes from the beginning to the end of septum formation (septation) and from the end of septum formation to the start of cell separation (cell separation) in the two strains (WT and *pbr1-8*) growing in the absence (Figure 2, control) or the presence of sublethal and lethal concentrations of the three echinocandins (Figure 3, Figure 4 and Figure 5). Depending on the strain and concentration of the echinocandin, different effects in the cytokinesis were induced: (1) cytokinesis was normal, with the elapsed times of septation and separation being similar to those of control cells (Table 3, boxes depicted in green); (2) cytokinesis was not blocked, but cell death occurred at the onset of cell separation (Table 3, boxes depicted in red); and (3) cytokinesis was blocked or delayed in the septum progression, and interrupted (or greatly delayed) the cell separation onset, and, thus, cell death did not occur (Table 3, boxes depicted in other colors). Thus, we analyzed how each echinocandin affects the cytokinesis progression (see below in the following subsection) and cell integrity (see below in the last section).

#### 2.2.1. Anidulafungin

Treatment of the WT strain with sublethal concentrations of anidulafungin for 3 h mainly induced an increase in the elapsed times of both septum formation and the onset of cell separation, without blocking cytokinesis progression (Table 3, Figure 3A, and Figure S1A). In contrast, the elapsed times for septation and cell separation onset in the *pbr1-8* mutant cells treated with sublethal concentrations of the drug were similar to those observed in the non-treated control cells (Table 3, Figure 3A, and Figure S1A). Interestingly, in both strains, lethal concentrations of anidulafungin arrested septum formation and consequently impeded the cell-separation onset (Table 3, Figure 3B, and Figure S1B). These results show that anidulafungin induces defects in the synthesis of β(1,3)-D-glucan that affect the progression of septation. These defects are also proportional to the antifungal concentration: low concentrations slow down septation, whereas higher concentrations completely block it. Thus, sublethal concentrations of the drug primarily induced two phenotypes in the WT strain; slower cytokinesis progression and cell death at the start of cell separation, which were similar to those described for Bgs4 depletion [30]. Additionally, the fact that both phenotypes were absent in the *pbr1-8* strain (Figure 3A and Figure S1A) indicates that lower concentrations of anidulafungin cause the sole inhibition of Bgs4. In contrast, the septation arrest observed in the presence of lethal concentrations of the drug was not corrected in the *pbr1-8* strain, indicating that these concentrations of the drug lead to the combined inhibition of Bgs4 together with Bgs1 and/or Bgs3 (Figure 3B). In agreement, septum ingression is similarly impaired when the function of Bgs1 is depleted or reduced in some mutant alleles [19,23,24,25].

#### 2.2.2. Caspofungin

Next, cytokinesis progression in the presence of caspofungin was examined. Sublethal concentrations of the drug resulted in a slight slowdown of the cytokinesis progression, with many WT cells dying at the onset of cell separation (Table 3, Figure 4A, WT, Type 1, and Figure S2A). After approximately 1.5 h of growth in the presence of the antifungal, many WT cells started to exhibit a progressive swelling of the growing pole, giving rise to cells with a bulb or drumstick appearance. Interestingly, due to a blockage of the onset of cell separation, most of these drumstick-like cells were still alive by the end of the 3 h treatment (Table 3, Figure 4A, WT, Type 2, and Figure S2A). The same drumstick-like cells with blocked separation were also observed in the presence of sublethal concentrations of both anidulafungin and micafungin (Table 3, Figures S1A and S3A, yellow arrows), as previously described for the echinocandin lipopeptide aculeacin A [32]. The *pbr1-8* mutation partially suppressed the phenotype of cell death at the start of cell separation, with many cells at the earliest treatment times exhibiting normal cytokinesis (Table 3, data not shown). However, most of the resistant cells that entered cytokinesis at later treatment times acquired the same drumstick appearance of the WT strain (Table 3, Figure 4A, and Figure S2A). Although lethal concentrations of caspofungin did not block the cytokinesis progression like anidulafungin (Figure 3B), the septum completion was extremely slowed down, and the cytokinesis was still incomplete by the end of the 3 h treatment (Table 3 and Figure 4A). The *pbr1-8* mutation partially suppressed the septum progression defects of cells exposed to higher doses of caspofungin (≥121.6 ± 26.5 min in the WT compared to 51.4 ± 8.4 min in the resistant strain), although, because of the blocked cell separation, cytokinesis progression was still very delayed and incomplete by the end of the 3 h treatment (Table 3 and Figure 4B). The fact that the *pbr1-8* mutation did not completely suppress the phenotypes observed in the WT strain suggests that caspofungin also acted against Bgs4 and Bgs1 and/or Bgs3, like anidulafungin. In agreement and similarly to anidulafungin, lethal concentrations of caspofungin led to an extremely slow cytokinesis development.

#### 2.2.3. Micafungin

In contrast to the other two echinocandins, the studies performed with micafungin showed that this drug solely inhibits the activity of Bgs4 at both sublethal and lethal concentrations (Table 3 and Figure 5). Thus, lower micafungin concentrations did not significatively affect cytokinesis progression, inducing a phenotype of cell death at the onset of cell separation (Table 3, Figure 5A and Figure S3A). At later treatment times, a few drumstick-like cells with blocked separation were also observed (Table 3 and Figure S3A, yellow arrows). Interestingly, both phenotypes were entirely suppressed in the resistant *pbr1-8* strain (Table 3, Figure 5A and Figure S3A). Similarly, lethal concentrations of the drug only caused a slight slowdown of the cytokinesis progression, with all the cells in cytokinesis dying at the cell separation onset. In these conditions, no drumstick-like cells were observed within the 3 h treatment (Table 3, Figure 5B and Figure S3B), suggesting that those drumstick-like cells may be the germ for the survivors observed after 24 h of treatment with sublethal concentrations of the three drugs. Again, non-cell death was observed in the treated cells of the *pbr1-8* strain, which also exhibited a normal cytokinesis progression (Table 3, Figure 5B and Figure S3B). It is noteworthy to mention that the cytokinesis phenotypes observed in the WT strain with lethal concentrations of micafungin were the opposite of those with anidulafungin and caspofungin. Additionally, while the *pbr1-8* mutation suppressed all the phenotypes caused by both sublethal and lethal concentrations of the micafungin, the mutation did not totally suppress the phenotypes caused by the other two echinocandins. All these results show that both sublethal and lethal concentrations of micafungin selectively inhibit Bgs4. In contrast, the other two echinocandins, Bgs1 and Bgs3, seem to not be affected by micafungin. This observation also might suggest that these two enzymes are not the targets for micafungin.

Currently, it is unknown why Bgs4 is susceptible to all the examined GS inhibitors, while Bgs1 and Bgs3 are susceptible to some inhibitors (anidulafungin and caspofungin) but not susceptible to others (micafungin, papulacandin B, and enfumafungin). Although Bgs1, Bgs3, and Bgs4 show high degrees of conservation (approximately 55% identity), they might still acquire different tertiary structures, making Bgs1 and Bgs3 less accessible to some echinocandins and more accessible to others. Another explanation might be due to changes in the lipid composition of the microdomains containing the GS subunits. Lipid composition impacts the structural and functional properties of the cell membranes, thus influencing the function of integral membrane proteins, such as the GS enzymes. Besides this, specific lipids may distribute asymmetrically between leaflets, originating different nanoscale domains along the plasma membrane [51]. Echinocandins are cyclic hexapeptides with a lipid side chain (Table 1) that is essential for their antibiotic activity and toxicity [52]. The lipid side chain is thought to interact with the outer leaflet of the plasma membrane containing the GS enzymes [45]. Thus, the differential susceptibility of the Bgs proteins to the echinocandins might be caused by differences in the organization and/or composition of the Bgs-containing lipid microenvironment. Consequently, it has been proposed that changes in the membrane lipids’ composition might modulate the interaction of echinocandins with the plasma membrane depending on the structure of the lipid side chain [53,54]. Alternatively, caspofungin and anidulafungin might affect the function of Bgs1 and/or Bgs3 through a secondary mechanism. Caspofungin and anidulafungin treatments induce a reduction in the levels of Bgs1 in the cleavage furrow (our unpublished results). In the budding yeast, the echinocandin B inhibits the localization of Spa2 [55], a cell polarity protein that regulates the localization of Chs2, which is responsible for the chitin synthesis of the primary septum [56]. Similarly, *S. pombe* Spa2 interacts with the F-BAR protein Cdc15 that is essential for the localization of Bgs1 to the division site [19,56].

### 2.3. Differential Effects of the Echinocandins in Cell Integrity

Next, the phenotypes of cell death, as well as the percentages corresponding to each phenotype, were analyzed in the time-lapse microscopy experiments described in the previous section. Depending on the effect of echinocandins in cytokinesis progression, differential levels of cell death were observed. Sublethal concentrations of any of the three antifungals did not block cytokinesis (Table 3), inducing death in 80–90% of the cells in the examined fields (Table 4). The cell death occurred either at the start of cell separation (Figure 3A, Figure 4A and Figure 5A) or during polarized growth in interphase (Figures S1A–S3A, and S4). Interestingly, these high percentages of initial death were not enough for arresting the cell growth (Table 2), probably because of the emergence of drumstick-like survivors at the later times of the 3 h treatment (Figures S1A–S3A, yellow arrows). Something similar is observed at longer times of Bgs4 depletion, where a small population of drumstick-like cells is also able to survive ([30], and our unpublished results). As expected, lethal concentrations also induced differential effects in cell integrity (Table 4). Both anidulafungin and caspofungin were primarily fungistatic and caused low percentages of cell death (22–27%) because they blocked the cell separation. In contrast, micafungin did not block cell separation, mainly being a fungicide and inducing high percentages of cell death (97%) (Table 4).

**Table 4:** Percentages and types of cell death observed in the time-lapses of WT and *pbr1-8* cells growing in the presence of sublethal (2 µg/mL) or lethal (20 µg/mL) concentrations of the three echinocandin drugs.

Drug (Concentration)	Strain	Number of Cells	Cell Death ^1^	Cell Lysis ^2^	Non-Cell Lysis ^3^
**Control (+DMSO)**	**WT**	*n = 30*	0 ^4^ (0) ^5^	0 ^4^ (0) ^5^	0 ^4^ (0) ^5^
	***pbr1-8***	*n = 33*	0 (0)	0 (0)	0 (0)
**Anidulafungin** **(2 µg/mL)**	**WT**	*n = 79*	89.9 (100)	65.2 (72.5)	24.7 (27.5)
	***pbr1-8***	*n = 61*	0 (0)	0 (0)	0 (0)
**Anidulafungin** **(20 µg/mL)**	**WT**	*n = 60*	21.7 (100)	13.3 (61.5)	8.4 (38.5)
	***pbr1-8***	*n = 45*	8.9 (100)	2.2 (25)	6.7 (75)
**Caspofungin** **(2 µg/mL)**	**WT**	*n = 96*	82.3 (100)	58.3 (70.9)	24 (29.1)
	***pbr1-8***	*n = 85*	22.4 (100)	5.9 (26.3)	16.5 (73.7)
**Caspofungin** **(20 µg/mL)**	**WT**	*n = 33*	27.3 (100)	12.1 (44.4)	15.2 (55.6)
	***pbr1-8***	*n = 64*	22.7 (100)	0 (0)	22.7 (100)
**Micafungin** **(2 µg/mL)**	**WT**	*n = 99*	80.3 (100)	13.6 (17)	69.7 (83)
	***pbr1-8***	*n = 36*	0 (0)	0 (0)	0 (0)
**Micafungin** **(20 µg/mL)**	**WT**	*n = 61*	97.5 (100)	97.5 (100)	0 (0)
	***pbr1-8***	*n = 46*	0 (0)	0 (0)	0 (0)

^1^ Cell death is the sum of the percentages of cell lysis and non-cell lysis. ^2^ Cells shrunk and died with cell breakage and cytoplasm leakage. ^3^ Cells shrunk and died without cell breakage and cytoplasm leakage. ^4^ Value is the percentage of dead cells with respect to the total number of visualized cells (*n*). ^5^ Value is the percentage of cell lysis or non-cell lysis with respect to the total number of dead cells.

As expected, the *pbr1-8* resistant mutation entirely suppressed the cell death caused by both sublethal and lethal concentrations of micafungin, reinforcing the finding that micafungin only inhibits Bgs4 activity (Table 4 and Table 5). Similarly, the *pbr1-8* mutation either suppressed or reduced the death caused by sublethal and lethal concentrations of anidulafungin, respectively (Table 4 and Table 5). In contrast, the *pbr1-8* mutation partially suppressed the death observed at lower concentrations (from 80% to 22%) but was largely ineffective with higher concentrations of caspofungin (22% in both WT and *pbr1-8*). Again, these results reinforce that caspofungin and anidulafungin inhibit several GS subunits to different degrees.

**Table 5:** Percentages of cell lysis either at interphase or at cell separation onset observed in the time-lapses of WT and *pbr1-8* cells growing in the presence of sublethal (2 µg/mL) or lethal (20 µg/mL) concentrations of the three echinocandin drugs.

Drug (Concentration)	Strain	Number of Cells	Total Cell Lysis ^1^	Lysis at Separation ^1^	Lysis at Interphase ^1^
**Control (+DMSO)**	**WT**	*n = 30*	0 ^2^ (0) ^3^	0 ^2^ (0) ^3^	0 ^2^ (0) ^3^
	***pbr1-8***	*n = 33*	0 (0)	0 (0)	0 (0)
**Anidulafungin** **(2 µg/mL)**	**WT**	*n = 79*	65.2 (72.5)	45.6 (50.7)	19.6 (21.8)
	***pbr1-8***	*n = 61*	0 (0)	0 (0)	0 (0)
**Anidulafungin** **(20 µg/mL)**	**WT**	*n = 60*	13.3 (61.5)	10.0 (46.1)	3.3 (15.4)
	***pbr1-8***	*n = 45*	2.2 (25)	0 (0)	2.2 (25)
**Caspofungin** **(2 µg/mL)**	**WT**	*n = 96*	58.3 (70.9)	40.6 (49.4)	17.7 (21.5)
	***pbr1-8***	*n = 85*	5.9 (26.3)	2.4 (10.5)	3.5 (15.8)
**Caspofungin** **(20 µg/mL)**	**WT**	*n = 33*	12.1 (44.4)	12.1 (44.4)	0 (0)
	***pbr1-8***	*n = 64*	0 (0)	0 (0)	0 (0)
**Micafungin** **(2 µg/mL)**	**WT**	*n = 99*	13.6 (17)	3.0 (3.8)	10.6 (13.2)
	***pbr1-8***	*n = 36*	0 (0)	0 (0)	0 (0)
**Micafungin** **(20 µg/mL)**	**WT**	*n = 61*	97.5 (100)	44.3 (45.4)	53.3 (54.6)
	***pbr1-8***	*n = 46*	0 (0)	0 (0)	0 (0)

Cell lysis is cell death accompanied by cell breakage and cytoplasm leakage. ^1^ Total cell lysis is the sum of the percentages of both cell lysis at interphase and at cell separation onset. ^2^ Value is the percentage of lysed cells with respect to the total number of cells (*n*). ^3^ Value is the percentage of lysed or non-lysed cells with respect to the total number of dead cells.

The three echinocandins induced two death phenotypes: death with cytoplasm leakage (Figures S1–S3, lysis, red arrows) and without cytoplasm leakage (Figures S1–S3, non-lysis, white arrows). Sublethal concentrations of anidulafungin and caspofungin mainly induced a lytic phenotype that mostly occurred at the onset of cell separation and was almost entirely suppressed in the *pbr1-8* strain (Table 4 and Table 5). In contrast, the cell death without an apparent leakage of the cytoplasm was still observed in the *pbr1-8* cells treated with caspofungin and was residual in the case of anidulafungin (Table 4 and Table 5). Intriguingly, the results of micafungin show a phenotypic difference in cell death that depends on the used concentration (Figure 5 and Figure S3). Sublethal concentrations of this antifungal induced, in the WT strain, a very high percentage of death without lysis (70%), while, with lethal concentrations, the totality of cell death involved the release of the cytoplasmic content (Table 3 and Table 5). Both death phenotypes were completely suppressed in the *pbr1-8* strain, indicating that both micafungin effects are due to the Bgs4 function. The phenotype of cell lysis is clearly the result of a localized rupture of the plasma membrane located under the weakened and/or reduced cell wall because of the reduction in the Bgs4-derived branched β(1,3)-D-glucan [19,30]. In contrast, death without lysis does not involve release of cytoplasmic content and might be due to a general alteration of plasma membrane permeability. The same phenotype of death without lysis was also observed in a few cells during Bgs4 depletion (our unpublished results) or when the function of the regulatory subunit of the GS complex, GTPase Rho1, is affected in the mutant allele *rho1-596* [57]. Endoplasmic reticulum (ER) stress in the budding yeast induces the permeabilization of the vacuolar membrane, causing cell acidification and death [58]. Interestingly, it has been described in several yeast species that cell wall stress activates an ER stress-like response that requires the entry of calcium and the function of the phosphatase calcineurin [57]. Thus, the cell damage caused by micafungin-inhibition of Bgs4 might activate a calcineurin-dependent ER stress-like response in fission yeast. In agreement, many mutants resistant to the calcineurin inhibitor FK506 exhibit hypersensitivity to micafungin [59]

## 3. Materials and Methods

### 3.1. Strains and Culture Conditions

The *S. pombe* strains examined in this study were isogenic to the WT strain h^-^ 972. The *pbr1-8* mutant was isolated by ethyl methane sulfonate mutagenesis and selection in the presence of 20 µg/mL papulacandin B [29]. The standard rich yeast growth medium (YES from “Yeast Extract with Supplements”) has been described previously [60]. Cell growth was monitored by measuring the A600 of early log-phase cell cultures in a Smart-Spec 3000 spectrophotometer (Bio-Rad, Hercules, CA, USA; A600 0.1 = 10^6^ cells/mL).

### 3.2. Antifungal Drugs and Susceptibility Assays

The echinocandins used in this study were generous gifts from Pfizer, New York, NY, USA (anidulafungin), Merck Sharp and Dohme, Kenilworth, NJ, USA (caspofungin), and Astellas Pharma, Chūō, Tokyo, Japan (micafungin). The echinocandins were kept at −20 °C in stock solution (10 mg/mL in DMSO) and assayed at the final concentrations specified in the text, tables, and figures. For micro-culture assays of a large number of samples, log-phase cultures grown in YES medium were diluted to a cell density of 5 × 10^5^ cells/mL (lower cell density) or 5 × 10^6^ cells/mL (higher cell density) in YES medium containing increasing concentrations of echinocandins (1, 2, 4, 10, 20, 40, and 80 µg/mL) or an equal volume of solvent (0.8% DMSO), which was the control cell culture. The cell cultures were grown in an orbital roller at 28 °C, and turbidity was examined after 24 h of growth. The MIC was the minimal concentration of echinocandin that induced a complete inhibition of the cell growth after 24 h of growth. The values were calculated from at least three independent experiments.

### 3.3. Microscopy Techniques and Data Analysis

Images of cells after 24 h of growth in the presence of the echinocandins (Figure 1) were directly obtained from the micro-cultures used for the susceptibility assays with a Nikon Eclipse 50i microscope, a Nikon Plan FLUOR 20 ×/0.45 objective, a Nikon Ds-Fi1 digital camera, and a Nikon Digital Sight DS-L2 control unit. Time-lapse imaging was performed as previously described [49]. A volume of 0.3 mL of logarithmic-phase cells grown in YES medium at 28 °C was collected at the cell density of 5 × 10^6^ cells/mL. The cells were gently centrifuged (1 min at 1000g), and the cell pellet was suspended in the same YES medium containing calcofluor white (CW) at a very low final concentration of 1.25 μg/mL together with 0.8% DMSO as control or the corresponding echinocandin at a final concentration of 2 µg/mL (sublethal, non-inhibitory) or 20 µg/mL (lethal, inhibitory), and placed in a well from a μ-Slide 8 well (80,821-Uncoated; Ibidi, Gräfelfing, Germany) previously covered with 5 μL of 1 mg/mL soybean lectin (L1395; Sigma-Aldrich, Burlington, MA, USA). All the time-lapse experiments were performed at 28 °C by acquiring epifluorescence and phase contrast cell images in single planes every 4 min and 1 × 1 binning on an inverted microscope (Olympus IX71) equipped with a PlanApo 100 ×/1.40 IX70 objective and a Personal DeltaVision system (Applied Precision LLC., Issaquah, WA, USA). Images were obtained using CoolSnap HQ2 monochrome camera (Photometrics, Tucson, AZ, USA) and softWoRx 5.5.0 release 6 imaging software (Applied Precision LLC.). Subsequently, CW time-lapse images were restored and corrected by 3D Deconvolution (conservative ratio, 10 iterations, and medium noise filtering) through soft-WoRx imaging software. Finally, images were processed with Image J (National Institutes of Health, Bethesda, MD, USA) and Adobe Photoshop software. All the time-lapse videos were repeated at least twice, and the data were calculated from at least two independent experiments.

## 4. Conclusions

Here, we have compared the cytokinesis and cell integrity processes in cells treated with sublethal and lethal concentrations of three echinocandin drugs: anidulafungin, caspofungin, and micafungin. We found that micafungin only inhibits activity due to Bgs4. Thus, and similarly to Bgs4 depletion, both sublethal and lethal concentrations of this drug primarily caused a phenotype of cell death in the WT that was entirely suppressed by the *bgs4^+^* mutation in *pbr1-8* cells. Our results indicate that all three echinocandins appear to act via Bgs4. In comparison, from the examination of septation and cell separation, it is evident that anidulafungin appears to affect, in some degree, the functions of Bgs1 and/or Bgs3, while caspofungin affects to a lower extent, and micafungin does not affect at all. To our knowledge, this is the first study analyzing, in detail, the progression and dynamics of cytokinesis in the presence of the echinocandin drugs. This experimental approximation has helped us identify that echinocandins induce differential effects in cytokinesis and cell integrity. Globally, these results show that caspofungin and anidulafungin are the most effective echinocandins against the resistant strain *pbr1-8*, probably because they combinedly affect the function of Bgs4 and other GS catalytic subunits.

## Figures and Tables

**Table 1:** Chemical structure of the echinocandins derivatives approved for therapeutic use (table adapted from [10]).

Chemical Structure	Compound (Commercial Name and Proprietary Company)
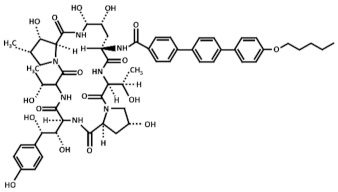	Anidulafungin (Eraxis^®^ or Ecalta^®^, Pfizer)
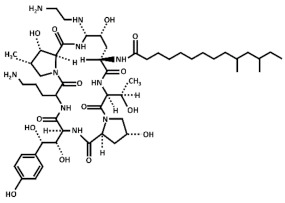	Caspofungin (Cancidas^®^, Merck, Sharp and Dhome, MSD)
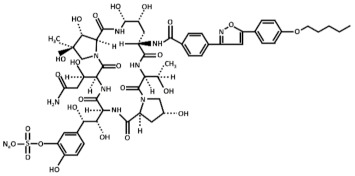	Micafungin (Mycamine^®^, Astellas Pharma)

**Table 2:** Susceptibilities of *S. pombe* wild-type and *pbr1-8* strains to echinocandins ^1^.

	Lower Cell Density ^2^	Higher Cell Density ^3^
**Strain**	**Anidulafungin MIC (µg/mL)**	
WT	4	10
*pbr1-8*	10	20
	**Caspofungin MIC (µg/mL)**	
WT	1	10
*pbr1-8*	2	40
	**Micafungin MIC (µg/mL)**	
WT	10	20
*pbr1-8*	>80	>80

^1^ The MIC was defined as the concentration of echinocandin at which growth of the corresponding *S. pombe* strain was completely inhibited after 24 h at 28 °C in YES medium. ^2^ Cells were diluted to a cell density of 5 × 10^5^ cells/mL and grown in microcultures of YES liquid medium with increasing concentrations (0, 1, 2, 4, 10, 20, 40, and 80 µg/mL) of echinocandins. ^3^ Cells were diluted to a cell density of 5 × 10^6^ cells/mL and grown in microcultures of YES liquid medium with increasing concentrations (0, 1, 2, 4, 10, 20, 40, and 80 µg/mL) of echinocandins.

**Table 3:** Cytokinesis phenotypes and elapsed times for septation and cell separation periods in *S. pombe* cells growing in culture chamber slides containing sublethal (2 µg/mL) or lethal (20 µg/mL) concentrations of the three echinocandins.

Drug (Concentration)	Cytokinesis Phenotype	Septation ^1^		Cell Separation ^2^	
		WT	*pbr1-8*	WT	*pbr1-8*
**Control (+DMSO)**	Normal cytokinesis	22.4 ± 2.4 *n = 30*	20.2 ± 1.6 *n = 33*	14.5 ± 2.4 *n = 27*	16.5 ± 3.4 *n = 33*
**Anidulafungin** **(2 µg/mL)**	Cell death ^3^	45.9 ± 10.5 *n = 25*	*n = 0*	36.3 ± 6.5 *n = 25*	*n = 0*
	Normal cytokinesis	*n = 0*	22.6 ± 2.3 *n = 60*	*n = 0*	21.4 ± 4.6 *n = 55*
	Slow septation Blocked separation ^4^	48.0 ± 0.0 *n = 1*	*n = 0*	≥84.0 ± 0.0 *n = 1*	*n = 0*
**Anidulafungin** **(20 µg/mL)**	Blocked cytokinesis ^5^	≥155.1 ± 24.9 *n = 23*	≥160.8 ± 15.4 *n = 31*	≥145.9 ± 36.1 *n = 7*	≥172.0 ± 0.0 *n = 1*
**Caspofungin** **(2 µg/mL)**	Cell death ^3^	31.0 ± 4.3 *n = 20*	32.0 ± 9.0 *n = 8*	32.4 ± 6.2 *n = 21*	26.0 ± 9.2 *n = 10*
	Normal cytokinesis	*n = 0*	24.3 ± 5.2 *n = 12*	*n = 0*	22.8 ± 4.9 *n = 17*
	Blocked separation	20.0 ± 2.9 *n = 14*	25.1 ± 4.3 *n = 11*	≥80.0 ± 26.2 *n = 14*	≥81.5 ± 36.5 *n = 11*
**Caspofungin** **(20 µg/mL)**	Extremely slow septation Blocked separation^4^	≥121.6 ± 26.5 *n = 24*	*n = 0*	≥ 75.3 ± 28.7 *n = 7*	≥105.1 ± 23.1 *n = 29*
	Slow septation	*n = 0*	51.4 ± 8.4 *n = 25*	*n = 0*	*n = 0*
**Micafungin** **(2 µg/mL)**	Cell death ^3^	22.8 ± 1.8 *n = 23*	*n = 0*	26.1 ± 6.8 *n = 31*	*n = 0*
	Normal cytokinesis	*n = 0*	20.1 ± 1.3 *n = 36*	*n = 0*	17.3 ± 2.7 *n = 33*
	Blocked separation	20.7 ± 1.8 *n = 6*	*n = 0*	≥52.0 ± 21.3 *n = 6*	*n = 0*
**Micafungin** **(20 µg/mL)**	Cell death^3^	27.6 ± 2.0 *n = 11*	*n = 0*	33.7 ± 4.3 *n = 12*	*n = 0*
	Normal cytokinesis	*n = 0*	19.8 ± 1.2 *n = 46*	*n = 0*	25.9 ± 5.0 *n = 39*

Values are minutes ± SD. ^1^ Elapsed time between septation onset and septation completion. ^2^ Elapsed time between septation completion and cell separation onset. ^3^ Cell death was at the onset of cell separation. ^4^ Separation onset is blocked or largely delayed. ^5^ Septation and cell separation are both blocked. *n* = number of cells that exhibit the corresponding phenotype within 3 h of imaging. Green boxes: normal cytokinesis; red boxes: cytokinesis with cell death at the onset of cell separation; and boxes in other colors: cytokinesis is either blocked or delayed.

## Data Availability

Data is contained within the article and Supplementary Material.

## Supplementary Materials

The following are available online at https://www.mdpi.com/article/10.3390/ph14121332/s1, Figure S1: Representative time-lapse field of WT and *pbr1-8* cells growing in the presence of sublethal and lethal concentrations of anidulafungin, Figure S2: Representative time-lapse field of WT and *pbr1-8* cells growing in the presence of sublethal and lethal concentrations of caspofungin, Figure S3: Representative time-lapse fields of WT and *pbr1-8* cells growing in the presence of sublethal and lethal concentrations of micafungin, Figure S4: Cell lysis and cytoplasm leakage during interphase in WT cells growing in the presence of the echinocandin drugs.

## References

[1] Bongomin F., Gago S., Oladele R.O., Denning D.W. (2017). Global and multi-national prevalence of fungal diseases-estimate precision. J. Fungi.

[2] Rudramurthy S.M., Hoenigl M., Meis J.F., Cornely O.A., Muthu V., Gangneux J.P., Perfect J., Chakrabarti A., ECMM, ISHAM (2021). ECMM/ISHAM recommendations for clinical management of COVID-19 associated mucormycosis in low- and middle-income countries. Mycoses.

[3] Limper A.H., Adenis A., Le T., Harrison T.S. (2017). Fungal infections in HIV/AIDS. Lancet Infect. Dis..

[4] LIFE Leading International Fungal Education: The Burden of Fungal Disease 2017. http://www.life-worldwide.org/media-centre/article/the-burden-of-fungal-disease-new-evidence-to-show-the-scale-of-the-problem.

[5] Perfect J.R. (2017). The antifungal pipeline: A reality check. Nat. Rev. Drug Discov..

[6] Ostrosky-Zeichner L., Casadevall A., Galgiani J.N., Odds F.C., Rex J.H. (2010). An insight into the antifungal pipeline: Selected new molecules and beyond. Nat. Rev. Drug Discov..

[7] Denning D.W., Bromley M.J. (2015). Infectious Disease. How to bolster the antifungal pipeline. Science.

[8] Arastehfar A., Gabaldón T., Garcia-Rubio R., Jenks J.D., Hoenigl M., Salzer H.J.F., Ilkit M., Lass-Florl C., Perlin D.S. (2020). Drug-resistant fungi: An emerging challenge threatening our limited antifungal armamentarium. Antibiotics.

[9] Ksiezopolska E., Schikora-Tamarit M.A., Beyer R., Nunez-Rodriguez J.C., Schuller C., Gabaldón T. (2021). Narrow mutational signatures drive acquisition of multidrug resistance in the fungal pathogen Candida glabrata. Curr. Biol..

[10] Cortés J.C.G., Curto M.A., Carvalho V.S.D., Pérez P., Ribas J.C. (2019). The fungal cell wall as a target for the development of new antifungal therapies. Biotechnol. Adv..

[11] Hopke A., Brown A.J.P., Hall R.A., Wheeler R.T. (2018). Dynamic Fungal Cell Wall Architecture in Stress Adaptation and Immune Evasion. Trends Microbiol..

[12] Georgopapadakou N.H., Tkacz J.S. (1995). The fungal cell wall as a drug target. Trends Microbiol..

[13] Curto M.A., Butassi E., Ribas J.C., Svetaz L.A., Cortés J.C.G. (2021). Natural products targeting the synthesis of β(1,3)-D-glucan and chitin of the fungal cell wall. Existing drugs and recent findings. Phytomedicine.

[14] Cabib E., Arroyo J. (2013). How carbohydrates sculpt cells: Chemical control of morphogenesis in the yeast cell wall. Nat. Rev. Microbiol..

[15] Lesage G., Bussey H. (2006). Cell wall assembly in *Saccharomyces cerevisiae*. Microbiol. Mol. Biol. Rev..

[16] Carvalho V.S.D., Gómez-Delgado L., Curto M.A., Moreno M.B., Pérez P., Ribas J.C., Cortés J.C.G. (2021). Analysis and application of a suite of recombinant endo-β(1,3)-D-glucanases for studying fungal cell walls. Microb. Cell Fact..

[17] Horiseberger M., Rosset J. (1977). Localization of α-galactomannan on the surface of *Schizosaccharomyces pombe* cells by scanning electron microscopy. Arch. Microbiol..

[18] Kreger D.R. (1954). Observations on cell walls of yeasts and some other fungi by x-ray diffraction and solubility tests. Biochim. Biophys. Acta.

[19] Cortés J.C.G., Ramos M., Osumi M., Pérez P., Ribas J.C. (2016). The Cell Biology of Fission Yeast Septation. Microbiol. Mol. Biol. Rev..

[20] Humbel B.M., Konomi M., Takagi T., Kamasawa N., Ishijima S.A., Osumi M. (2001). In situ localization of β-glucans in the cell wall of *Schizosaccharomyces pombe*. Yeast.

[21] Liu J., Balasubramanian M.K. (2001). 1,3-β-Glucan synthase: A useful target for antifungal drugs. Curr. Drug Targets-Infect. Disord..

[22] Latge J.P. (2007). The cell wall: A carbohydrate armour for the fungal cell. Mol. Microbiol..

[23] Le Goff X., Woollard A., Simanis V. (1999). Analysis of the *cps1* gene provides evidence for a septation checkpoint in *Schizosaccharomyces pombe*. Mol. Gen. Genet..

[24] Liu J., Wang H., Balasubramanian M.K. (2000). A checkpoint that monitors cytokinesis in *Schizosaccharomyces pombe*. J. Cell Sci..

[25] Ramos M., Cortés J.C.G., Sato M., Rincón S.A., Moreno M.B., Clemente-Ramos J.A., Osumi M., Pérez P., Ribas J.C. (2019). Two *S. pombe* septation phases differ in ingression rate, septum structure, and response to F-actin loss. J. Cell Biol..

[26] Liu J., Tang X., Wang H., Balasubramanian M. (2000). Bgs2p, a 1,3-β-glucan synthase subunit, is essential for maturation of ascospore wall in *Schizosaccharomyces pombe*. FEBS Lett..

[27] Roncero C., Sánchez Y. (2010). Cell separation and the maintenance of cell integrity during cytokinesis in yeast: The assembly of a septum. Yeast.

[28] Martín V., García B., Carnero E., Durán A., Sánchez Y. (2003). Bgs3p, a putative 1,3-β-glucan synthase subunit, is required for cell wall assembly in *Schizosaccharomyces pombe*. Eukaryot. Cell.

[29] Martins I.M., Cortés J.C.G., Muñoz J., Moreno M.B., Ramos M., Clemente-Ramos J.A., Durán A., Ribas J.C. (2011). Differential activities of three families of specific β(1,3)glucan synthase inhibitors in wild-type and resistant strains of fission yeast. J. Biol. Chem..

[30] Muñoz J., Cortés J.C.G., Sipiczki M., Ramos M., Clemente-Ramos J.A., Moreno M.B., Martins I.M., Pérez P., Ribas J.C. (2013). Extracellular cell wall β(1,3)glucan is required to couple septation to actomyosin ring contraction. J. Cell Biol..

[31] Johnson B.F. (1968). Lysis of yeast cell walls induced by 2-deoxyglucose at their sites of glucan synthesis. J. Bacteriol..

[32] Miyata M., Kitamura J., Miyata H. (1980). Lysis of growing fissin-yeast cells induced by aculeacin A, a new antifungal antibiotic. Arch. Microbiol..

[33] Cassone A., Mason R.E., Kerridge D. (1981). Lysis of growing yeast-form cells of *Candida albicans* by echinocandin: A cytological study. Sabouraudia.

[34] Yamaguchi H., Hiratani T., Baba M., Osumi M. (1985). Effect of aculeacin A, a wall-active antibiotic, on synthesis of the yeast cell wall. Microbiol. Immunol..

[35] Vicente M.F., Basilio A., Cabello A., Peláez F. (2003). Microbial natural products as a source of antifungals. Clin. Microbiol. Infect..

[36] Suwunnakorn S., Wakabayashi H., Kordalewska M., Perlin D.S., Rustchenko E. (2018). FKS2 and FKS3 Genes of Opportunistic Human Pathogen *Candida albicans* Influence Echinocandin Susceptibility. Antimicrob. Agents Chemother..

[37] Pappas P.G., Kauffman C.A., Andes D.R., Clancy C.J., Marr K.A., Ostrosky-Zeichner L., Reboli A.C., Schuster M.G., Vázquez J.A., Walsh T.J. (2016). Clinical Practice Guideline for the Management of Candidiasis: 2016 Update by the Infectious Diseases Society of America. Clin. Infect. Dis..

[38] Perlin D.S. (2015). Echinocandin Resistance in *Candida*. Clin. Infect. Dis..

[39] Pfaller M.A., Messer S.A., Jones R.N., Castanheira M. (2015). Antifungal susceptibilities of *Candida*, *Cryptococcus neoformans* and *Aspergillus fumigatus* from the Asia and Western Pacific region: Data from the SENTRY antifungal surveillance program (2010–2012). J. Antibiot..

[40] Johnson M.E., Katiyar S.K., Edlind T.D. (2011). New Fks hot spot for acquired echinocandin resistance in Saccharomyces cerevisiae and its contribution to intrinsic resistance of Scedosporium species. Antimicrob. Agents Chemother..

[41] Sawistowska-Schroder E.T., Kerridge D., Perry H. (1984). Echinocandin inhibition of 1,3-β-D-glucan synthase from *Candida albicans*. FEBS Lett..

[42] Douglas C.M., Marrinan J.A., Li W., Kurtz M.B. (1994). A *Saccharomyces cerevisiae* mutant with echinocandin-resistant 1,3-β−D-glucan synthase. J. Bacteriol..

[43] Taft C.S., Stark T., Selitrennikoff C.P. (1988). Cilofungin (LY121019) inhibits *Candida albicans* (1-3)-β-D-glucan synthase activity. Antimicrob. Agents Chemother..

[44] Perlin D.S. (2007). Resistance to echinocandin-class antifungal drugs. Drug Resist. Updates.

[45] Johnson M.E., Edlind T.D. (2012). Topological and mutational analysis of *Saccharomyces cerevisiae* Fks1. Eukaryot. Cell.

[46] Jiménez-Ortigosa C., Jiang J., Chen M., Kuang X., Healey K.R., Castellano P., Boparai N., Ludtke S.J., Perlin D.S., Dai W. (2021). Preliminary structural elucidation of β-(1,3)-glucan synthase from *Candida glabrata* using cryo-electron tomography. J. Fungi.

[47] García R., Itto-Nakama K., Rodríguez-Pena J.M., Chen X., Sanz A.B., de Lorenzo A., Pavon-Verges M., Kubo K., Ohnuki S., Nombela C. (2021). Poacic acid, a β-1,3-glucan-binding antifungal agent, inhibits cell-wall remodeling and activates transcriptional responses regulated by the cell-wall integrity and high-osmolarity glycerol pathways in yeast. FASEB J..

[48] Roncero C., Celador R., Sánchez N., García P., Sánchez Y. (2021). The role of the cell integrity pathway in septum assembly in yeast. J. Fungi.

[49] Cortés J.C.G., Ramos M., Konomi M., Barragán I., Moreno M.B., Alcaide-Gavilán M., Moreno S., Osumi M., Pérez P., Ribas J.C. (2018). Specific detection of fission yeast primary septum reveals septum and cleavage furrow ingression during early anaphase independent of mitosis completion. PLoS Genet..

[50] Sipiczki M. (2007). Splitting of the fission yeast septum. FEMS Yeast Res..

[51] Makarova M., Peter M., Balogh G., Glatz A., MacRae J.I., Lopez Mora N., Booth P., Makeyev E., Vigh L., Oliferenko S. (2020). Delineating the rules for structural adaptation of membrane-associated proteins to evolutionary changes in membrane lipidome. Curr. Biol..

[52] Boeck L.D., Fukuda D.S., Abbott B.J., Debono M. (1989). Deacylation of echinocandin B by *Actinoplanes utahensis*. J. Antibiot..

[53] Healey K.R., Katiyar S.K., Raj S., Edlind T.D. (2012). CRS-MIS in *Candida glabrata*: Sphingolipids modulate echinocandin-Fks interaction. Mol. Microbiol..

[54] Satish S., Jiménez-Ortigosa C., Zhao Y., Lee M.H., Dolgov E., Kruger T., Park S., Denning D.W., Kniemeyer O., Brakhage A.A. (2019). Stress-induced changes in the lipid microenvironment of β-(1,3)-D-glucan synthase cause clinically important echinocandin resistance in *Aspergillus fumigatus*. mBio.

[55] Okada H., Ohnuki S., Roncero C., Konopka J.B., Ohya Y. (2014). Distinct roles of cell wall biogenesis in yeast morphogenesis as revealed by multivariate analysis of high-dimensional morphometric data. Mol. Biol. Cell.

[56] Foltman M., Filali-Mouncef Y., Crespo D., Sánchez-Díaz A. (2018). Cell polarity protein Spa2 coordinates Chs2 incorporation at the division site in budding yeast. PLoS Genet..

[57] Viana R.A., Pinar M., Soto T., Coll P.M., Cansado J., Pérez P. (2013). Negative functional interaction between cell integrity MAPK pathway and Rho1 GTPase in fission yeast. Genetics.

[58] Kim H., Kim A., Cunningham K.W. (2012). Vacuolar H^+^-ATPase (V-ATPase) promotes vacuolar membrane permeabilization and nonapoptotic death in stressed yeast. J. Biol. Chem..

[59] Ma Y., Jiang W., Liu Q., Ryuko S., Kuno T. (2011). Genome-wide screening for genes associated with FK506 sensitivity in fission yeast. PLoS ONE.

[60] Alfa C., Fantes P., Hyams J., McLeod M., Warbrick E. (1993). Experiments with Fission Yeast: A Laboratory Course Manual.

