# Supplementary material for: Echinocandin Drugs Induce Differential Effects in Cytokinesis Progression and Cell Integrity
Source: Pharmaceuticals (Basel). 2021 Dec 20;14(12):1332. doi: 10.3390/ph14121332 (PMC8706178; doi:10.3390/ph14121332)
Supplement: Supplementary file 1 [file pharmaceuticals-14-01332-s001.zip › pharmaceuticals-1494787-supplementary.pdf]

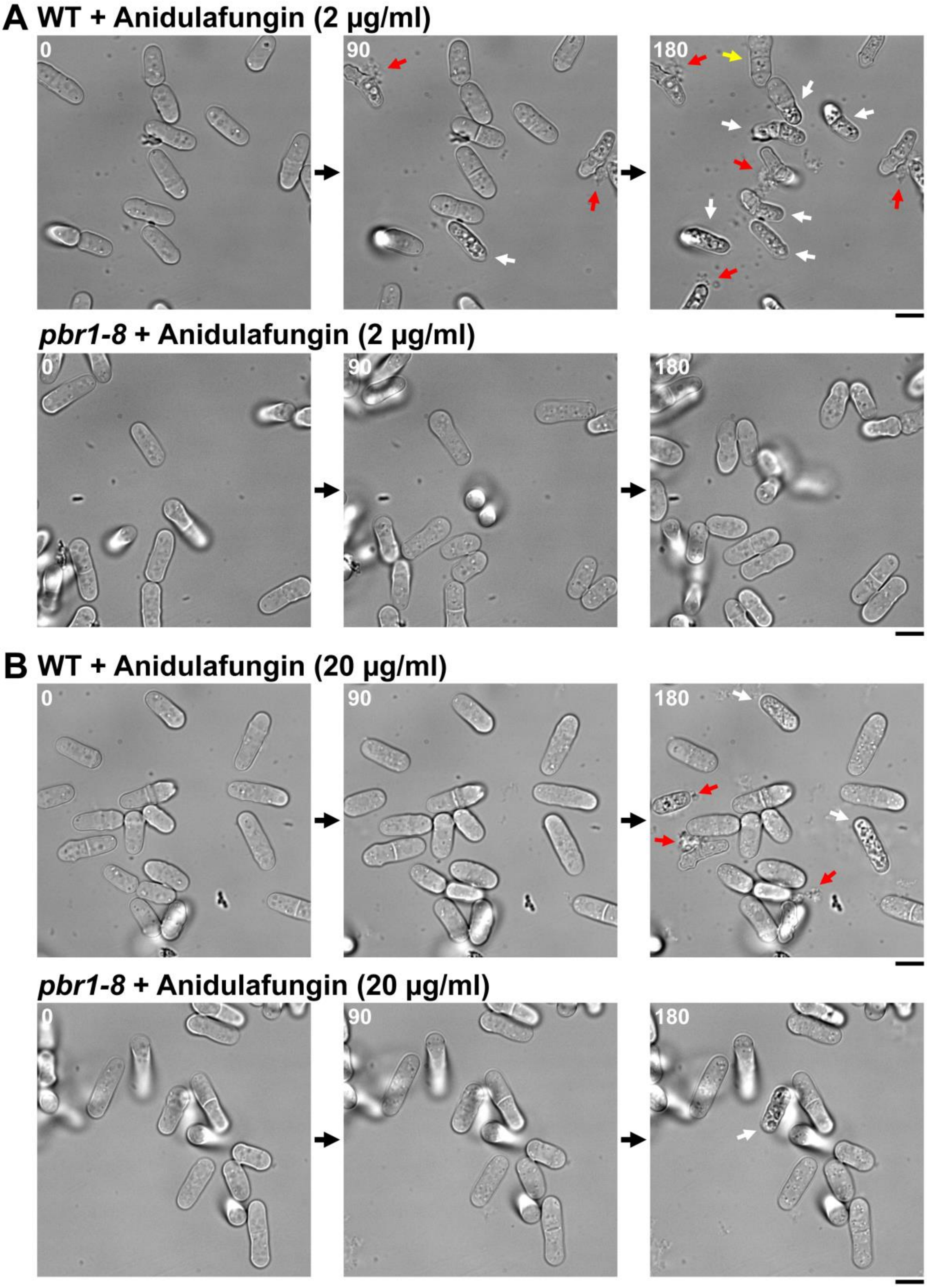

Figure S1. Representative time-lapse field of WT and *pbr1-8* cells growing in the presence of sublethal and lethal concentrations of anidulafungin. The indicated strains were grown and imaged as in Figure 2 in the presence of sublethal ((**A**), 2 µg/ml) or lethal ((**B**), 20 µg/ml) concentrations of the drug. Only the initial (time = 0 minutes), middle (time = 90 minutes), and final (time = 180 minutes) phase contrast time-lapse frames are depicted. The data of this figure are developed in Tables 3–5. Arrows: white, death without apparent cytoplasm leakage (non-cell lysis); red, death with cytoplasm leakage (cell lysis). Scale bars, 10 µm.

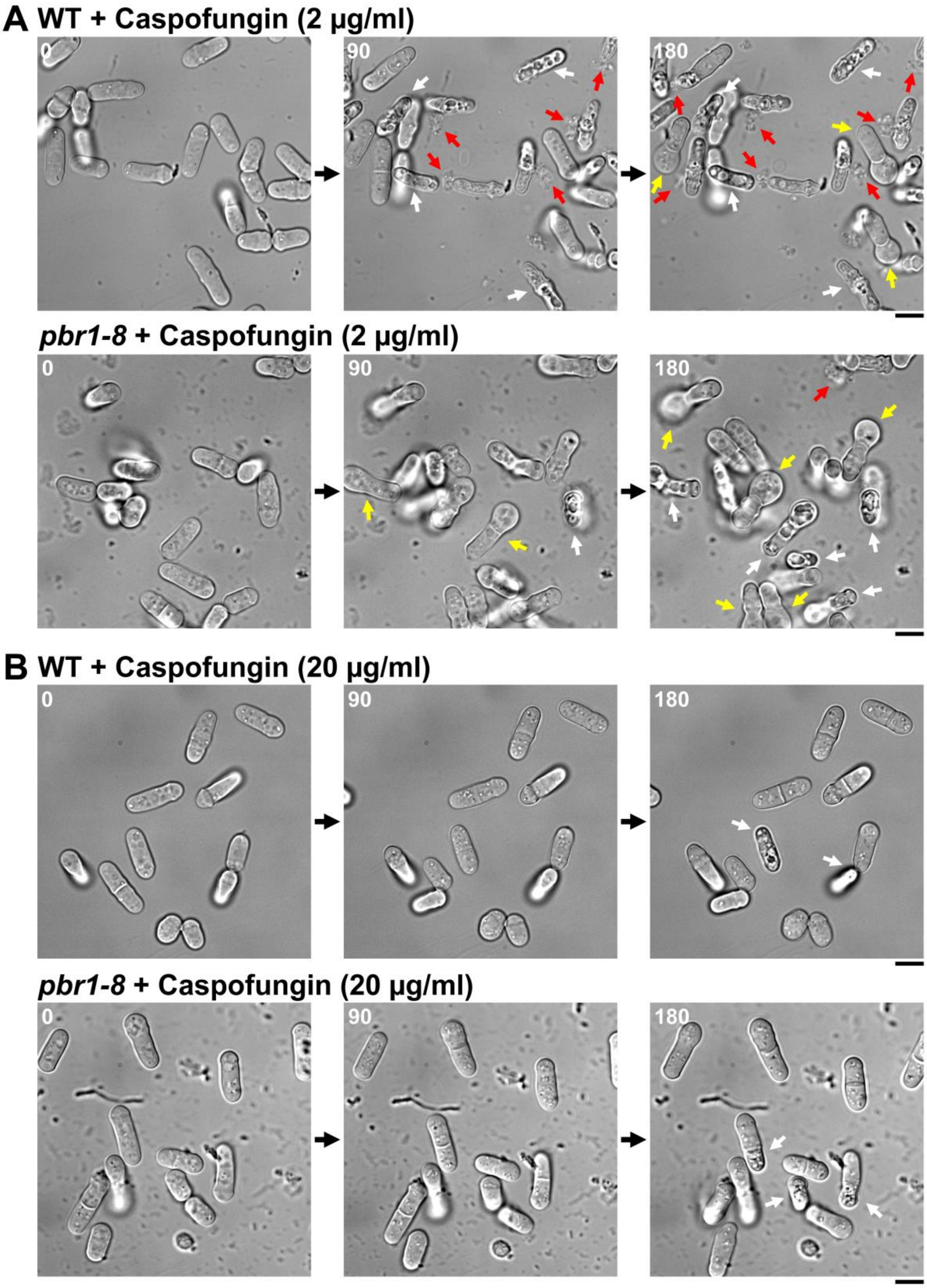

Figure S2. Representative time-lapse field of WT and *pbr1-8* cells growing in the presence of sublethal and lethal concentrations of caspofungin. The indicated strains were grown and imaged as in Figure 2 in the presence of sublethal ((**A**), 2 μg/ml) or lethal ((**B**), 20 μg/ml) concentrations of the drug. The data of this figure are developed in Tables 3–5. Time-lapse frames and arrows are as in figure S1. Scale bars, 10 μm.

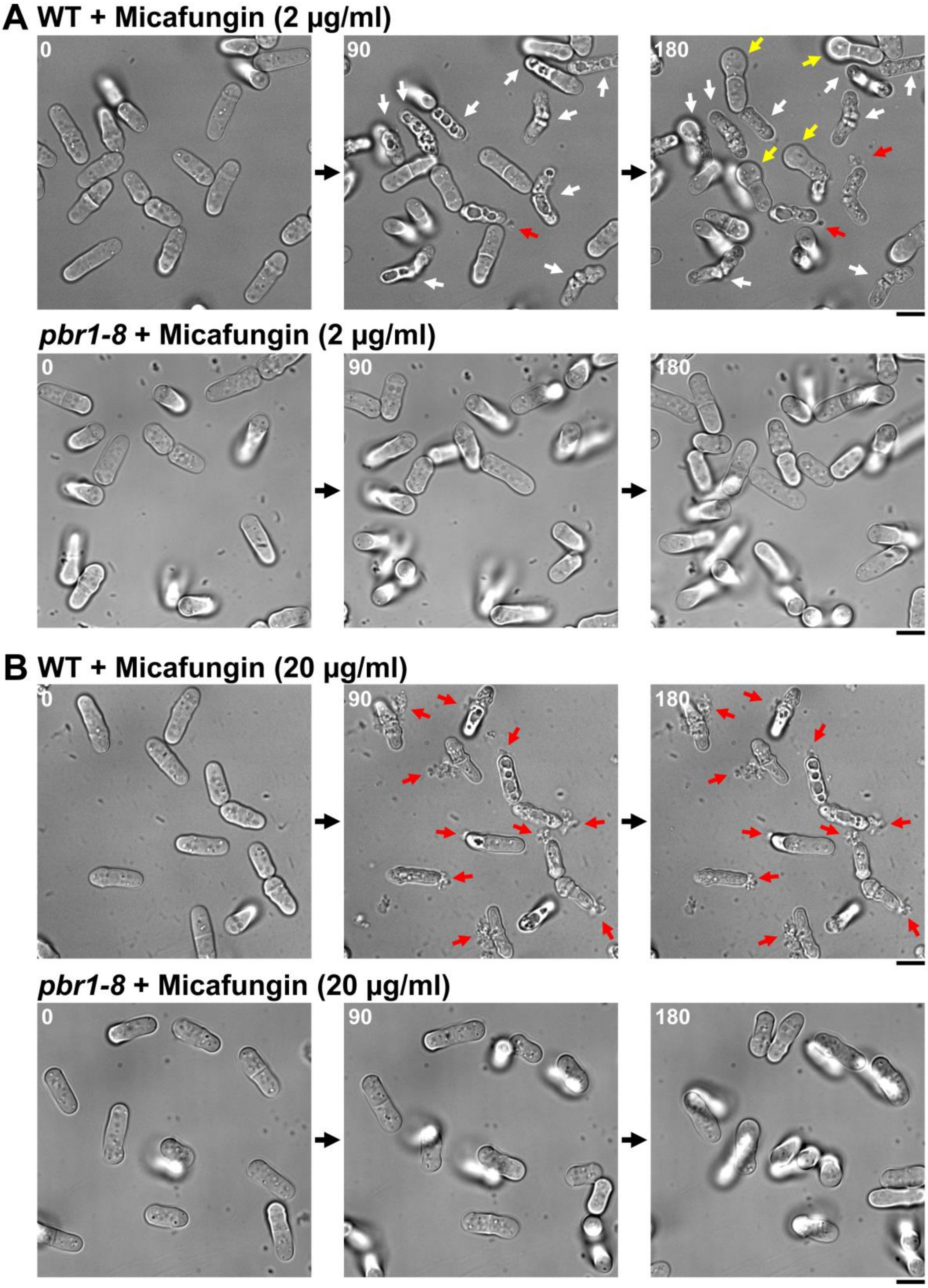

Figure S3. Representative time-lapse fields of WT and *pbr1-8* cells growing in the presence of sublethal and lethal concentrations of micafungin. The indicated strains were grown and imaged as in Figure 2 in the presence of sublethal ((**A**), 2 µg/ml) or lethal ((**B**), 20 µg/ml) concentrations of the drug. The data of this figure are developed in Tables 3–5. Time-lapse frames and arrows are as in figure S1. Scale bars, 10 µm.

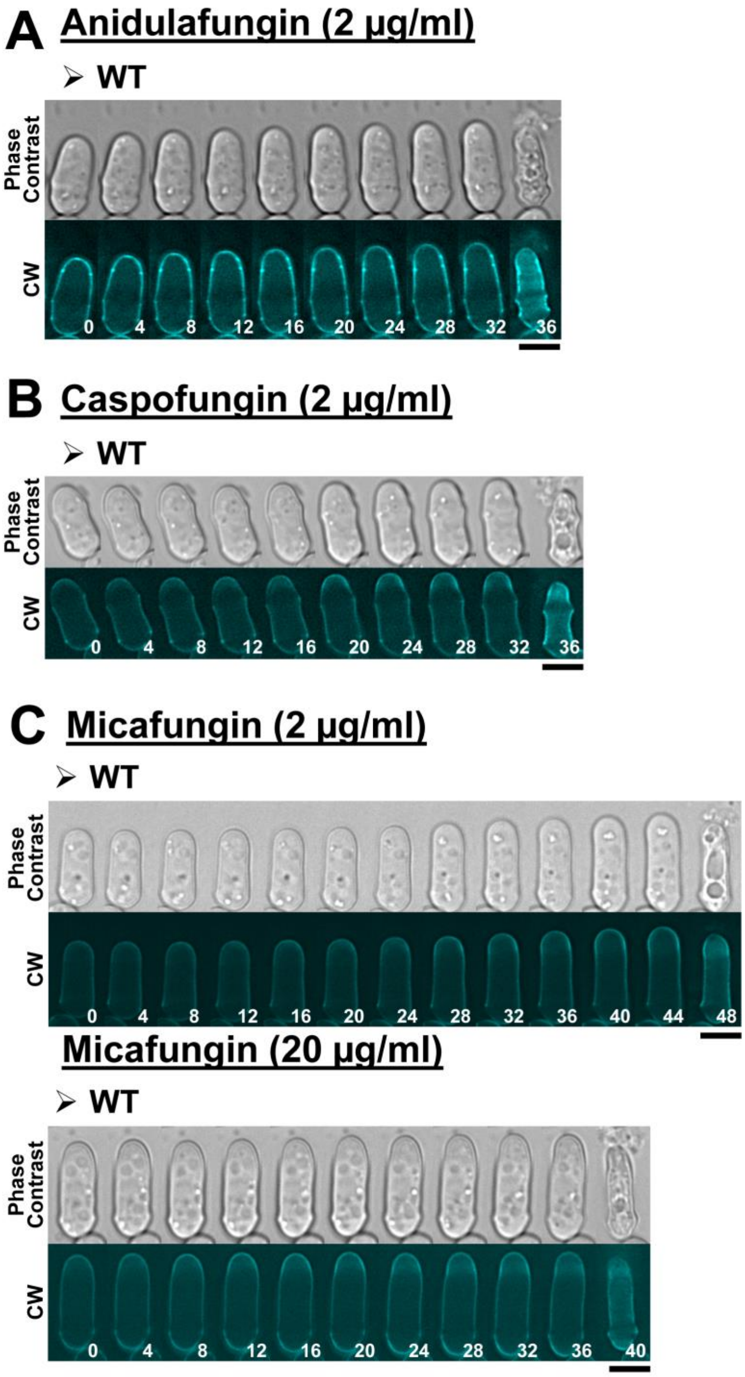

Figure S4. Cell lysis and cytoplasm leakage during interphase in WT cells growing in the presence of the echinocandin drugs. The indicated strains were grown and imaged as in Figure 2 in the presence of the indicated concentrations of anidulafungin (A), caspofungin (B) and micafungin (C). The data of this figure are developed in Table 4 and 5. Scale bars, 5 μm.
